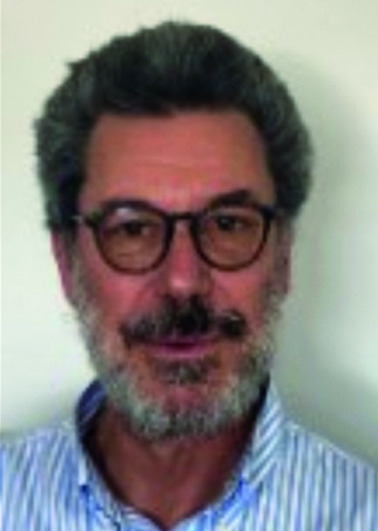# GDF15 Analogues Acting as GFRAL Ligands

**DOI:** 10.1002/cmdc.202400961

**Published:** 2025-02-17

**Authors:** Andrea Di Santo, Livio Tarchi, Gianluca Villa, Giovanni Castellini, Valdo Ricca, Roberta Squecco, Anna Maria Papini, Feliciana Real‐Fernandez, Paolo Rovero

**Affiliations:** ^1^ Department of Neuroscience Psychology Pharmacology and Infant Health Interdepartmental Research Unit of Peptide and Protein Chemistry and Biology University of Florence Via Ugo Schiff, 6, Sesto Fiorentino FI, 50019 Italy; ^2^ Department of Health Science Psychiatry Unit University of Florence, Largo Brambilla 3 Florence FI, 50134 Italy; ^3^ Department of Health Science Anesthesiology Unit University of Florence, Largo Brambilla 3 Florence FI, 50134 Italy; ^4^ Department of Experimental and Clinical Medicine Section of Physiological Sciences University of Florence, Viale Morgagni 63 Florence FI, 50134 Italy; ^5^ Department of Chemistry “Ugo Schiff” Interdepartmental Research Unit of Peptide and Protein Chemistry and Biology University of Florence via della Lastruccia, 3–13, Sesto Fiorentino FI, 50019 Italy; ^6^ Institute of Chemistry of Organometallic Compounds – National, Research Council of Italy (ICCOM-CNR) Via Madonna del Piano, 10, Sesto Fiorentino FI, 50019 Florence Italy

**Keywords:** Cytokines, Growth factors, Peptides, Proteins, Receptors

## Abstract

Growth differentiation factor 15 (GDF15) is a TGF‐β superfamily member involved in diverse physiological and pathological processes. It is expressed in various tissues and its circulating levels rise during exercise, aging, pregnancy, and conditions such as cancer, cardiovascular disease, and infections. The biological activities of GDF15, including anorexia and cachexia, are primarily mediated through the GFRAL receptor, localized in the brainstem and functioning via RET co‐receptor recruitment. This signaling is crucial for energy homeostasis and nausea induction. Recent studies suggest a broader GFRAL distribution, potentially explaining GDF15′s distinct roles. These findings sparked interest in leveraging GDF15‐GFRAL pathways for therapeutic development. Two primary strategies include GDF15 analogues as GFRAL agonists for obesity treatment and GDF15‐derived peptides as antagonists to counteract cancer‐induced cachexia and related disorders. This review highlights advancements in understanding GDF15‐GFRAL signaling and its implications, summarizing bioactive GDF15‐derived molecules, their pharmacological applications, and offering insights into novel treatment avenues for GDF15‐associated conditions.

## Graphical abstract

Considering the great importance of the GDF15/GRAL/RET signalling pathway, this review focuses on GDF15‐derived analogues able to act either as GFRAL agonists or antagonists, investigating their possible pharmacological applications in the treatment of GDF15‐related conditions, such as obesity and diabetes (agonist), or cancer‐induced nausea and cachexia (antagonist).

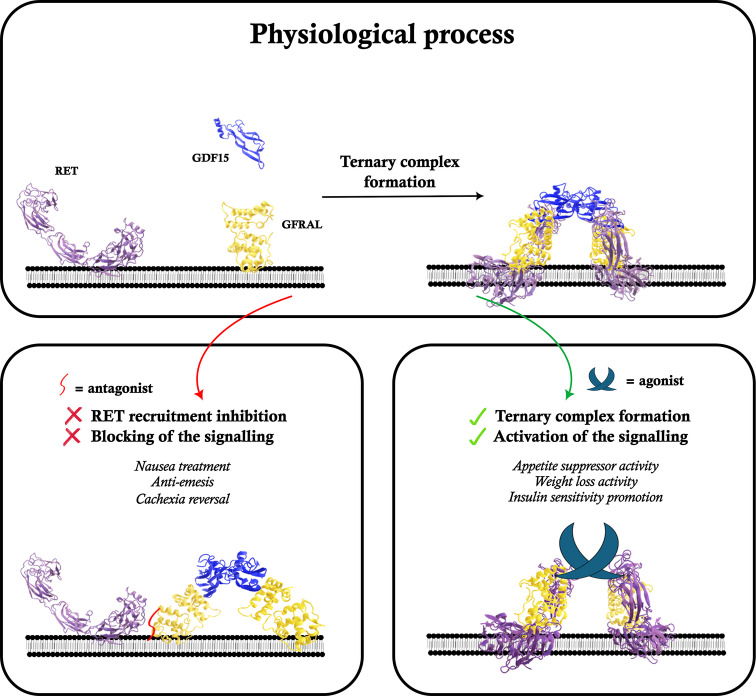

## 1 Introduction

**Figure 1:**
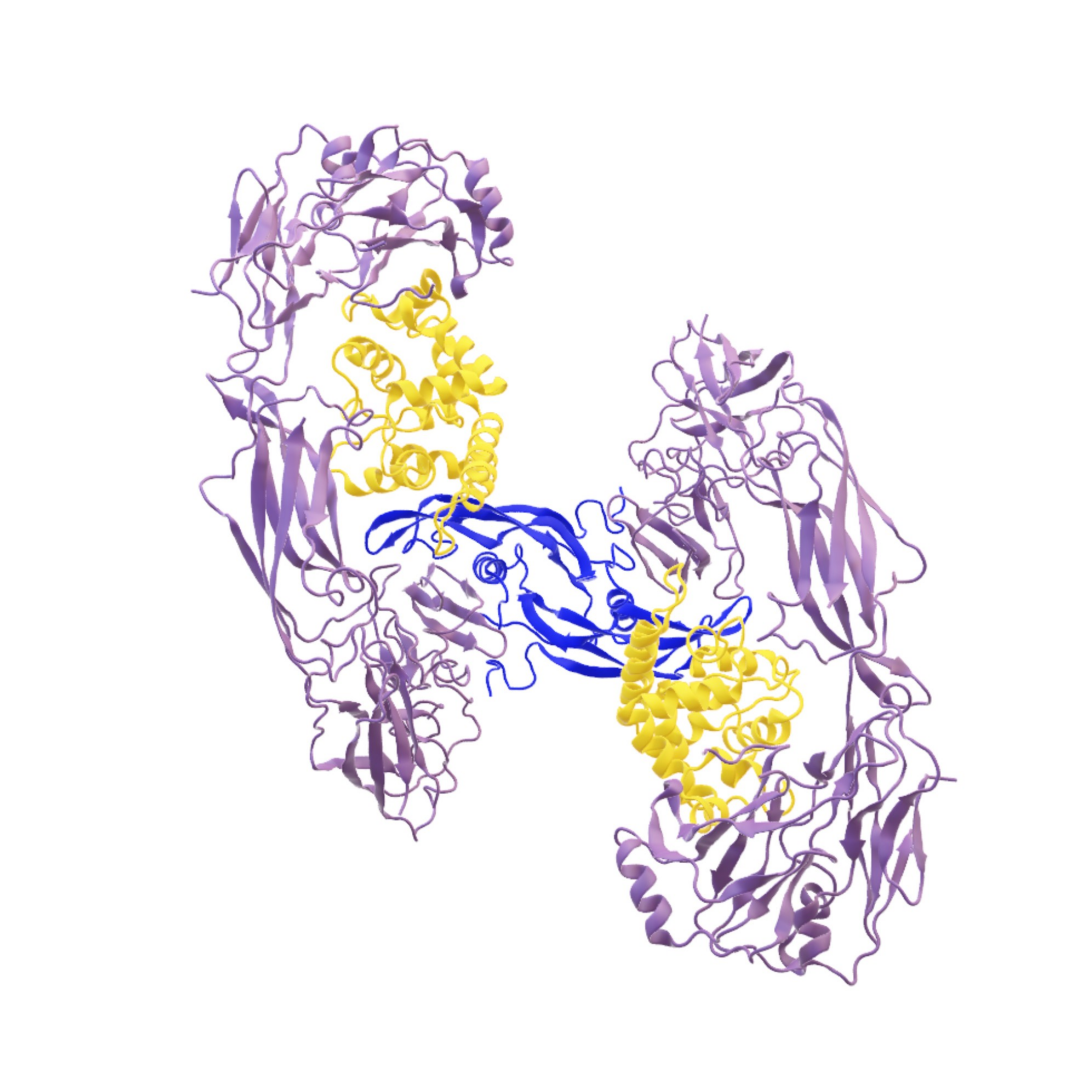
Cryo‐EM structure of extracellular dimeric complex of GDF15/GFRAL/RET (PDB: 6Q2J). Blue: GDF‐15; yellow: GFRAL; lilac: RET.

Growth differentiation factor 15 (GDF15) is a 25 kDa dimeric cysteine knot protein belonging to the transforming growth factor‐beta (TGF‐β) superfamily, firstly described in 1997.[^1^, ^2^] As other TGF‐β superfamily members, GDF15 is involved in different biological functions and it is expressed in a variety tissues such as lung, liver, placenta, and pancreas.^[3]^ Moreover, GDF‐15 serum levels have been found to be increased under physiological conditions such as exercise, aging, or pregnancy,[^4^, ^5^, ^6^] but also to increase within different clinical conditions, *e. g*., cancer, cardiovascular diseases, kidney disease, and viral infections.[^7^, ^8^, ^9^, ^10^] Increased circulating levels of GDF15 during such conditions are related to anorexia (loss of appetite) and cachexia (extreme weight loss and muscle wasting).^[11]^ The molecular mechanism driving such effects remained unclear until 2017, when the GDNF family receptor α‐like (GFRAL) was found as principal receptor of GDF15 and described to be exclusively expressed in the brainstem area postrema (AP) and nucleus tractus solitarii (NTS) in mice. High levels of GDF15 activate this receptor, thus causing anorexia and cachexia.[^12^, ^13^] The apparent discrepancy between the widespread expression and pleiotropic actions of GDF15,^[14]^ and the restricted localization of GRAL in NTS/AP, lead to hypothesize a more extensive receptor distribution, maybe at low but biologically sufficient levels. Indeed, in addition to the classically reported localization, GFRAL expression has recently been described as widely distributed both in the central nervous system and peripheral tissues,^[15]^ potentially elucidating how GDF15 might exhibit different effects under different physio pathological conditions. To the present day, GDF15 activity on GFRAL receptors has mainly been described in relation to the control of energy homeostasis and induction of nausea and emesis.[^13^, ^16^, ^17^, ^18^] GFRAL belongs to the glial cell line‐derived neurotrophic factor (GDNF) receptor family. As other GDNF receptors, signalling occurs through the recruitment of co‐receptor RET, a tyrosine kinase receptor, with the formation of a ternary complex (here: GDF15/GFRAL/RET) (Figure 1).[^13^, ^16^, ^19^, ^20^, ^21^, ^22^] The key role of this ternary complex is confirmed, for example, by the fact that the inactivation of GFRAL through metalloproteinases or anti‐GFRAL antibodies has been reported to negatively regulate GDF15/GFRAL signalling and suppress GDF15‐induced anorexia,[^16^, ^23^] or that GFRAL knock‐out mice have been found to be resistant to weight loss, nausea, and cachexia induced by chemotherapy.^[20]^

Recently, different studies have investigated the molecular mechanism of action of GDF15, in particular its interaction with GFRAL and RET, and how GDF15 could be exploited to develop new pharmaceuticals to treat different conditions associated with excessive or deficient GDF15 expression. In this context, two different pathways are envisaged: 1) taking advantage of its proprieties as an appetite suppressor, a weight loss promoter, and an agent against insulin resistance to build a pharmacologically administered GDF15 analogue acting as a GFRAL agonist to treat obesity, diabetes and prediabetes, or 2) to block GFRAL receptor signalling through GDF15 analogues acting as GFRAL antagonists, for the treatment of diseases in which GDF15 is highly expressed, such as cancer‐induced anorexia or cachexia. In this review both these approaches will be addressed, summarizing studies that describe bioactive GDF15 derived molecules and their possible pharmacological applications.

## 2 GFRAL Agonists

**Figure 2:**
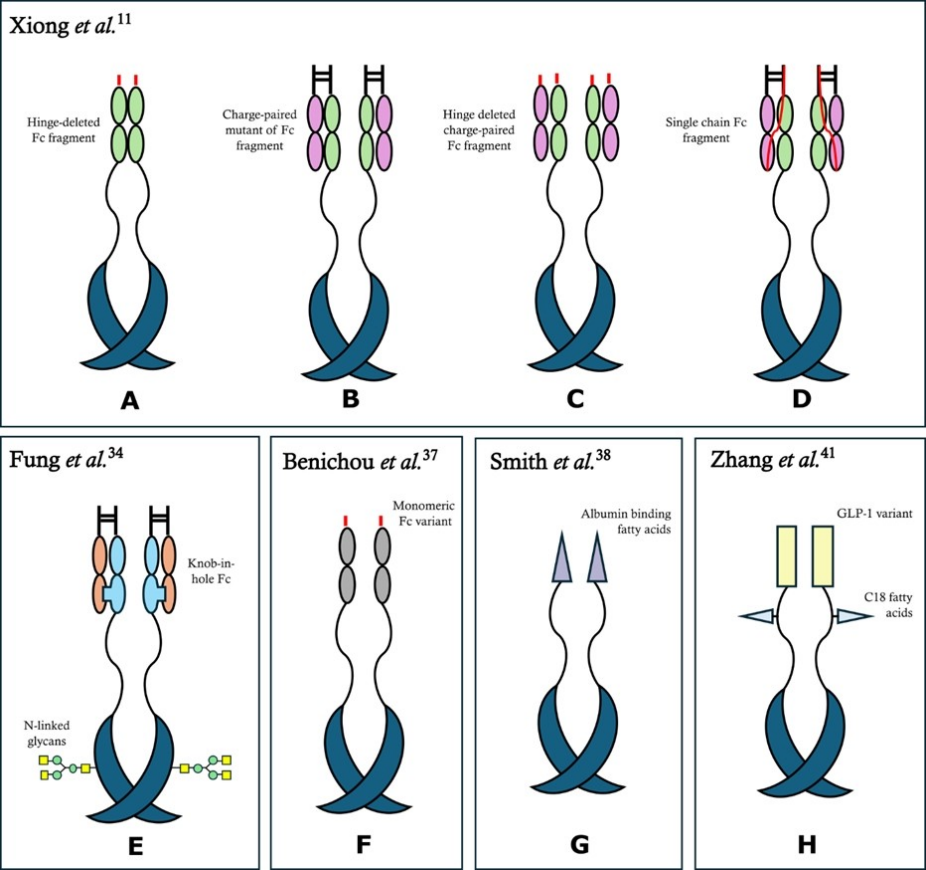
Structures of GFRAL agonist. GDF15 variants, represented in blue, are fused with different moieties: (A) one hinge‐deleted Fc fragment, (B) two charge‐paired mutants of Fc fragment, (C) two hinge deleted charge‐paired Fc fragments, (D) two single‐chain Fc fragments, (E) two knob‐in‐hole Fc, (F) two monomeric Fc variants, (G) two albumin binding fatty acids, (H) two GLP‐1analogues with C18 fatty acids on the linker. All the linkers connecting GDF15 variants with the different moieties are peptide sequences, as indicated in the text, except for (G), in which the linker is polyethylene glycol.

The appetite suppressing function of GDF15 highlighted the possibility of using it to reduce body weight.^[24]^ Considering that the signalling pathway of GDF15/GFRAL/RET is highly conservated in rodents and non‐human primates, various *in vivo* studies confirmed this opportunity.[^24^, ^25^, ^26^] For instance, the administration of GDF15 (through viral vectors or recombinant protein injections) in animal models (genetic obese ob/ob mouse) improved overall metabolic parameters, reducing food intake and body weight.[^26^, ^27^] Moreover, transgenic mice overexpressing GDF15 from birth were insensitive from glucose intolerance, diet‐induced obesity, and hepatic steatosis.[^20^, ^28^] However, these promising outcomes collided with the pharmacokinetic and physicochemical properties of wildtype GDF15, which constrained its development as a therapeutic. In fact, extracellular GDF15 has an extremely low stability, due to proteolytic cleavage, and a high tendency to aggregate, leading to a quite short half‐life (approximately 3 hours in mice and non‐human primates).[^26^, ^29^] Thus, the development of GDF15 analogues started from the improvement of its stability and its half‐life, in order to allow dose frequency reduction. A common strategy to improve pharmacokinetics properties is the generation of recombinant protein in which GDF15 is fused with moieties that could improve its half‐life. For instance, the fusion with Human Serum Albumin, HSA‐GDF15 protein (also known as Compound H, CpdH), has been found to produce robust weight loss in obese non‐human primates with improved pharmacokinetics properties.^[30]^ Another described approach involved the use of the Fc domain of IgG antibody, as reported by Xiong *et al*., investigating the possibility of fuse GDF15 with different Fc fragments.^[26]^ Firstly, Xiong *et al*. identified the suitable fusing site of GDF15, choosing the N‐terminus, since the analysis of its structure showed as the C‐terminus of the protein is not accessible for fusion. A direct fusion of GDF15 and Fc was challenging due to distance mismatch, so the flexible amino acid linker (G_4_S)_4_ was inserted, thus allowing the access to the GDF15 receptor to the canonical type I and type II receptor binding sites. The first selected fusion protein was a modified Fc, with deleted hinge region and bearing two copies of GDF15 (Figure 2A). However, despite being active in the ob/ob mouse food intake assay, this molecule was obtained in a very poor yield of ca 1 mg per litre of culture. To overcome this relevant problem, the second fusion protein was designed with only one GDF15 subunit per Fc dimer, incorporating a set of complementary charges in the CH3 domains of the Fc region (Figure 2B). This modification reduced Fc CH_3_ interface constraints, leading to an increase in protein yield (270 mg per litre of culture) but causing an increase in terms of ED_50_ from 2.1 μg/kg to 112 μg/kg. However, the activity was re‐established truncating the N‐terminal hinge region of Fc (ED_50_ 7.8 μg/kg), maintaining a good protein yield (Figure 2C). Thus, for *in vivo* investigations in mice and cynomolgus monkeys, the last fusion protein and the single chain Fc version (ED_50_ 18.4 μg/kg and yield 90 mg per litre of culture) were chosen (Figure 2D). Weekly treatment with both analogues in diet induced obese (DIO) mice and obese cynomolgus monkeys improved metabolic parameters, demonstrating a strong effect in lowering body weight.^[26]^

Another attempt to improve the physicochemical properties of GFRAL agonists has been represented by N‐glycosylation. N‐glycan additions to a protein may increase solubility, reduce aggregation, shielding hydrophobic regions, and also reduce enzymatic stability, shielding protease cleavage sites.[^31^, ^32^, ^33^] Using a structure‐based, rational design approach, Fung *et al*. combined N‐glycosylation of GDF15 with Fc functionalization through a knob‐into‐hole Fc technology to obtain a fusion protein with improved therapeutic properties (Figure 2E).^[34]^ In particular, each GDF15 unit of an homodimer was fused, via the flexible linker (G_4_S)_4_, to the N‐terminal of a knob‐Fc, paired with a co‐expressed hole Fc. This approach was chosen because of its ability to reduce the formation of aggregates, as compared to other Fc formats, such as IgG Fc‐GDF15 homodimer and a hole‐Fc‐GDF15/knob‐Fc‐GDF15 heterodimer. The following step was the selection of sites for N‐glycosylation, by introduction into the GDF15 peptide sequence of NxT motifs, which naturally undergo glycosylation during protein expression, thus yielding high site‐specific N‐glycosylation. Using the crystal structure of GDF15‐GFRAL complex (PDB 5VZ4), N‐glycosylations were introduced without compromising GFRAL receptor binding sites and aiming to shield known protease cleavage sites.^[20]^ In fact, GDF15 is reported to be cleaved by PCSK family proteases and mature GDF15 contains one well‐characterized cleavage site at R2 (resulting in a 112‐mer peptide) and a putative one at R4.[^2^, ^35^] Since the cleavage at both these sites may lead to the removal of the Fc moiety, a part of the mutants set was designed to protect the protein from these specific cleavages. Regarding R2 site, it was simply removed by the deletion of the first two residues, while the masking of R4 cleavage site was afforded through two different mutations. These two mutations were: i) A3 N/N5T substitution (Mutant 2) and ii) R4 N/G6T substitution (Mutant 3), which both showed greater *in vitro* serum stability, as compared to wild type Fc‐GDF15. Moreover, the crystal structure of GDF15‐GFRAL complex (PDB 5VZ4) offered the opportunity to insert single point mutations to improve the binding affinity of GDF15 to GFRAL.^[20]^ Using a sequence tolerance prediction algorithm, three mutants were designed: Mutants A (L36H) and B (L36R) introduced hydrogen bonding interactions, while Mutant C (V98I) was designed to fill a hydrophobic void at the binding interface. These single point mutations were singularly introduced in the two mutants that should block the cleavage site R4: mutant 2 (A3 N/N5T) and 3 (R4 N/G6T). These mutants, containing either an N‐glycosylation (2 o 3) or an amino acid substitution (A, B, or C), were expressed using the transient Expi293 cell system – except for Mutant 2B which was not expressed through this system, and therefore was dropped from the study. Mutant 2 C was found to be the most efficacious in inducing weight loss for wild type mice, featuring a two‐fold improvement in GFRAL binding activity (as measured through SPR). However, despite these improvements in GFRAL binding activity, Mutant 2 C did not necessarily result in better *in vivo* functional activity. Instead, Mutant 3B showed a three‐fold increase in GFRAL binding affinity, longer circulating lifetime, and significantly higher serum concentration. This high level of serum concentration was hypothesized to be caused by the binding to additional receptors (such as murine splice variant GRAL‐B) that may cause a localized Fc‐GDF15 reservoir *in vivo*.[^13^, ^16^, ^20^, ^36^] Despite clinical studies would be required to evaluate the potentialities of these mutants (in particular, Mutant 2 C, characterized by improved efficacy, and Mutant 3B, showing good half‐life), as here described, current research has already highlighted potential engineering candidates of GDF15 analogues acting as GFRAL agonists of clinical interest.

Nonetheless, the lack of clinical studies regarding GDF15 analogues acting as GFRAL agonists is the biggest limitation in the evaluation of their therapeutic potential. Up to now, only two analogues have been tested in humans: LY3463251, developed by Eli Lilly, in phase I clinical study, and MBL949, developed by Novartis, which reached phase II clinical study.[^37^, ^38^] LY3463251 is a recombinant protein composed by a monomeric variant of human IgG_4_, a rigid linker, and a truncated biologically active human GDF15 (Figure 2F). The monomeric Fc variant lacked the hinge region sequence ESKYGPPCPPCP to guarantee the formation of monomeric IgG_4_ variant and presented the substitutions F173Q and Y175E to disrupt the CH_3_/CH_3_ association. CH_2_ Ala231, Pro232, and Lys447 were also deleted to improve chemical stability. The C‐terminus was coupled through a rigid linker (GGGGAPAPAPAPAPAPAPAPAPAPGGGG) with the N‐terminus of truncated human GDF15 (from Gly200 to Ile308), leading to a covalently linked mature GDF15 homodimer formed through an interchain disulfide bond. The pairing with the hinge‐less IgG_4_ Fc allowed GDF15 folding and dimerization increased protein half‐life, while also leading to an improvement in term of production yield. LY3463251 caused a reduction in food intake and body weight in rodents and non‐human primates. However, these promising results were not reflected during the phase I study, where only modest effects were observed. Reduction of body weight over a 12‐weeks treatment period (−3 % versus placebo) was found not to be clinically significant, while the reduction in food intake was observed in humans too. However, in this clinical study, dose‐dependent nausea and emesis were observed. The authors discussed whether these adverse effects would also be associated, in turn, with weight loss, however, considering the results derived from different studies on GDF15 and the preclinical investigations on obese cynomolgus monkeys, nausea was not found to be correlated with weight loss. To be noted, the reduction in appetite observed in the cohort was independent from the insurgence of nausea or emesis, further strengthening the hypothesis that GDF15 can independently promote weight loss irrespectively of its central effect on AP/NTS neurons.^[37]^ The first clinical trial of a GDF15 analogue, however, showed important limitations and raised even more questions about this important molecule. The promising results emerging from preclinical investigations were not confirmed. It should be noted that the study was carried out during the COVID‐19 pandemic, so some planned analyses were not completed. For instance, the dose administrated was not as high as the level administrated to non‐human primates and this could explain why a significant weight loss was not observed despite the loss of appetite. To be noted, however, a dose increase was not fully attainable considering the adverse effects observed. Another aspect that the authors noted was the unexpected weight gain of the control group, that questioned about the fact that the participants may have been exposed to food and/or physical activity changes during the pandemic, thus impacting the results of the clinical study. From a pharmacological point of view, GDF15 therapeutics may show different central nervous system penetration for different species, *e. g*., humans compared to rodents and monkeys, thus leading to a different availability of the compound for GFRAL receptors. A reduction of the interaction with GFRAL receptors may also be caused by other possible factors that have not been yet fully investigated. For example, there may be unrecognized inhibitors of GDF15 that could prevent its binding to GFRAL or RET. Alternatively, obesity‐associated human‐specific changes in GDF15/GFRAL/RET complex signalling could be present, reducing its efficacy. Moreover, GDF15 acts in different ways on a variety of tissues, exhibiting pleiotropy and a hormetic potential. For instance, GDF15 also induces cortisol production, which is associated to an increase in appetite and weight gain, potentially explaining how GDF15 analogues may drive opposite results to the desired effects.^[39]^

Another GDF15 analogue acting as a GFRAL agonist is MBL949. MBL949 is a recombinant human GDF15 dimer conjugated to one or two albumin binding fatty acids via a short and flexible polyethylene glycol linker (Figure 2G). In animal models, the fatty acids conjugation extended the circulating half‐life from hours to days. The efficacy of fatty acid conjugation was hypothesized based on extrapolation from glucagon‐like peptide 1 (GLP1) therapeutics, in which acylated GLP‐1 analogues showed higher efficacy compared to the non‐acylated ones.^[40]^ Despite weight loss effects observed in all animal species, the same effects were not reported in humans, even though the occurrence of nausea as a pharmacodynamic marker of efficacious dosing was observed. Pharmacokinetic phase 1 data following single injection demonstrated a prolonged half‐life, supporting biweekly dosing, and further investigation suggested that a repeated dose administration could lead to weight loss effects, as observed in pre‐clinical studies. However, in the phase 2 study, results in terms of weight loss were mild, and the mean weight loss was −1.6 kg at week 16, providing no motivation for further investigations, considering dose‐dependent side effects such as gastrointestinal discomfort. Similarly to LY3463251, promising preclinical results did not fully translate to humans, despite the different molecular construct of MBL949. This may be caused by the dimensions of the two proteins (76 kDa for LY3463251 and 27 kDa for MBL949) that prevent their reach to the hindbrain area, in which receptors driving anorexia and emesis are located. This hypothesis of an effect of protein dimension acquires further significance considering that effective acylated GLP‐1 analogues feature a molecular weight around 5 kDa. Dose‐limiting tolerability also suggests that this pathway is less dominant in humans compared to other species, questioning if there is an issue generalizability of results from animals to humans.^[38]^

The concept of fusing GDF15 to another protein fragment may be easily extended to proteins with similar pharmacological properties, as demonstrated by Zhang *et al*.^[41]^ Zhang and colleagues fused GDF15 and a GLP‐1 analogues to obtain a fusion protein potentially exerting the anti‐obesity properties of both molecules (Figure 2H). The C‐terminus of a GLP‐1 derived peptide was fused, through a flexible peptide linker (from 4 up to 120 residues), to the N‐terminus of a GDF15 variant. Preliminary *in vitro* tests allowed to evaluate the optimal length of the linker: higher efficacies were found for longer linkers, however, for manufacturing reasons, the 80‐residues linker was chosen. Further engineering involving fatty acids conjugations gave the fusion protein half‐life extension. The best site to attach C18 fatty acid was found being in the middle of the linkers, as far as possible from both the active sites of GDF15 and GLP‐1. The resulted fusion protein, QL1005, was likely to have a dimer form, similar to native GDF15, which allowed an effective GDF15 signalling without a compromise to GLP‐1 activity. This balanced effect was confirmed by the dual activation of both GDF15 and GLP‐1 receptor *in vivo*, despite this had not been anticipated by the first protein design. The singular activity of the two moieties was also studied through two QL1005 variants, in which either GDF15 or GLP‐1 functionalities were knocked out, whose *in vivo* activity was demonstrated being comparable, and, more importantly, reduced by half compared to QL1005. When tested in mice and monkeys, QL1005 showed dose dependent weight loss effects, even at lower dose levels versus comparator semaglutide, highlighting the great potentiality of this type of fusion protein, which, however, remains to be confirmed in humans.^[41]^

## 3 GFRAL Antagonists

As previously mentioned, GFRAL could also be targeted with an antagonist, to inhibit GDF15/GFRAL signalling, and for the treatment of different conditions such as cachexia, anorexia, or chemotherapy‐induced nausea and vomiting (CINV).[^42^, ^43^, ^44^] Despite previous attempts to treat obesity through GFRAL agonists, the studies on antagonists are lagging behind their agonist counterpart, and interest by pharmacological companies seem to be less pronounced. Considering that in contrast to agonists, antagonists do not need to recruit both GFRAL and RET, pharmacological research focused on molecules able to interact only with GFRAL, thus blocking the formation of GDF15/GFRAL/RET complexes, and therefore the GDF15 downstream signalling pathway.

Lee *et al*. hypothesized that an anti‐GFRAL antibody could interfere with the formation of the ternary complex. Therefore, A11, a fully human anti‐GFRAL antibody, was selected *via* biopanning from a human combinatorial antibody phage library. Initial *in vitro* assays showed the specificity of the interaction of A11 with human GFRAL, and that this interaction occurred at the GFRAL/RET interface, without compromising the binding of GDF15 with the receptor. Moreover, A11 was administrated *in vivo* to mice in which cachexia was previously induced by cis‐platin, resulting in an amelioration of cachectic symptoms, also in melanoma‐bearing mice.^[45]^

**Figure 3:**
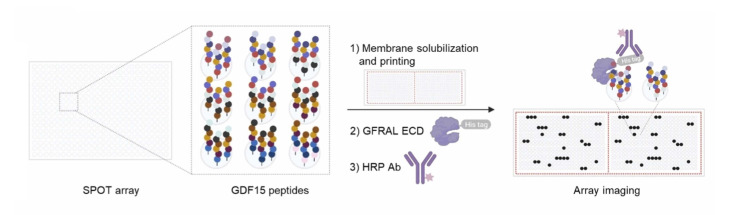
Schematic representation of a SPOT array. GDF15 derived peptides immobilized on the plate are screened against His‐tagged extracellular domain (ECD) of GFRAL. Horseradish peroxidase (HRP) conjugated 6xHis antibody allowed the detection using a chromogenic substance. Black spots represent GDF15 fragments bound to GFRAL ECD. Reproduced from Ref. [46].

A different approach stemmed from the observation that instead of exploiting unrelated molecular species, such as an antibody, it may be more interesting to start from the ligand, in this case GDF15, looking for specific regions of the protein able to bind to the receptor. Once identified, stand‐alone peptides could be synthesized. These should, theoretically, bind to GFRAL, thus inhibiting the formation of the complex with GDF15 and RET. The investigation from “peptide point of view” may start, as Alexopoulou *et al*. did, from a first screening of the full series of GDF15 overlapping fragments (epitope mapping) to find the sequences that better interacts with GFRAL extra‐cellular domain (ECD).^[46]^ Using the SPOT arrays technology (Figure 3), 95 GDF15 derived 15‐mer peptides, with a shift of one residue between each sequence, were synthesized directly onto a cellulose membrane, suitable for subsequent solid‐phase test. From this first screening, two sequences showed strong binding proprieties for GFRAL‐ECD: 88 (^287^KTDTGVSLQTYDDLL^301^) and 89 (^288^TDTGVSLQTYDDLLA^302^), both part of the binding interface formed by the C‐terminal hairpin of GDF15 and the D2 domain of GFRAL.^[20]^ Thereafter, a second SPOT array focused on the C‐hairpin sequence (residues 273–308) and longer peptides (from 16 to 23 residues) were synthesized, maintaining the one residue shift. What emerged was a shared overlapping sequence in the complementary β‐strands of the GDF15 C‐hairpin (residues 279–301) and the two most active sequences, 195 (^283^VLIQKTDTGVSLQTYDDLL^301^) and 253 (^279^YNPMVLIQKTDTGVSLQTYDDLL^301^), were tested in monoclonal GFRAL HEK cells transiently expressing the co‐receptor RET. However, both sequences showed only weak inhibition properties. One last library of non‐immobilized peptides, comprising 192 sequences from GDF region 201–308 of different lengths (from 26 up to 34 residues), with a two amino acids shift, was synthesized through solid phase peptide synthesis (substituting oxidation‐prone methionine with norleucine, represented by X in the one‐letter code sequences, to optimize synthesis steps). *In vitro* tests confirmed the good inhibitory of peptides derived from the C‐terminal region of GDF15 (271–304), in particular peptides 311 (^271^APCCVPASYNPXVLIQKTDTGVSLQTYDDLLAKD^304^) and 348 (^273^CCVPASYNPXVLIQKTDTGVSLQTYDDLLAKD^304^). Interestingly, the peptide 387, corresponding to 311, but lacking two N‐terminal cysteine residues (^275^VPASYNPXVLIQKTDTGVSLQTYDDLLAKD^304^), showed a loss of activity. This observation led the authors to hypothesize that the efficacy of 311 and 348 may derive from spontaneous oxidative dimerization, trough the formation of an intermolecular disulfide bridge. However, an analogue of 348 in which Cys was replaced by Ser (an isostere residue, unable to form disulfides) retained similar inhibition proprieties, refuting this hypothesis. Sequence 348, once purified, showed the best GFRAL inhibition activity (EC_50_=19.1±6.6 μM) and this was supposed to be caused by the disruption of both GDF15/RET and GDF15/GFRAL interfaces. Structural studies confirmed that the residues of peptide 348 were involved in the binding interface between a hydrophobic pocket located in the D2 domain of GFRAL ECD and the C‐hairpin of GDF15. In particular, Val283 and Ile285, both present in the peptide, had been previously demonstrated being fundamental for GDF15/GFRAL interaction.^[20]^ Additionally, the cryo‐EM structure of the ternary complex GDF15/GFRAL/RET revealed that the GDF15/RET interface site was opposite to GDF15/GFRAL binding site, resulting in a “sandwich” with GDF15 in the middle. Furthermore, it occurred between the C‐terminal cysteine rich domain of RET and N‐ and C‐hairpin loops of GDF15 through hydrophobic interactions, in which Tyr297 played a key role.^[21]^ This study was the first systematic investigation about the inhibition of the formation of the ternary complex GDF15/GFRAL/RET using peptides reproducing GDF15 binding epitopes as protein‐protein interaction modulators, obtained screening the whole GDF15 sequence. However, despite the fact that one peptide was found exhibiting inhibition activity, deeper investigations on the sequence are essential, starting from the determination of binding affinity to the receptors, up to the study of its pharmacological proprieties, trying to find out a proper candidate as GDF15 antagonist.^[46]^

**Figure 4:**
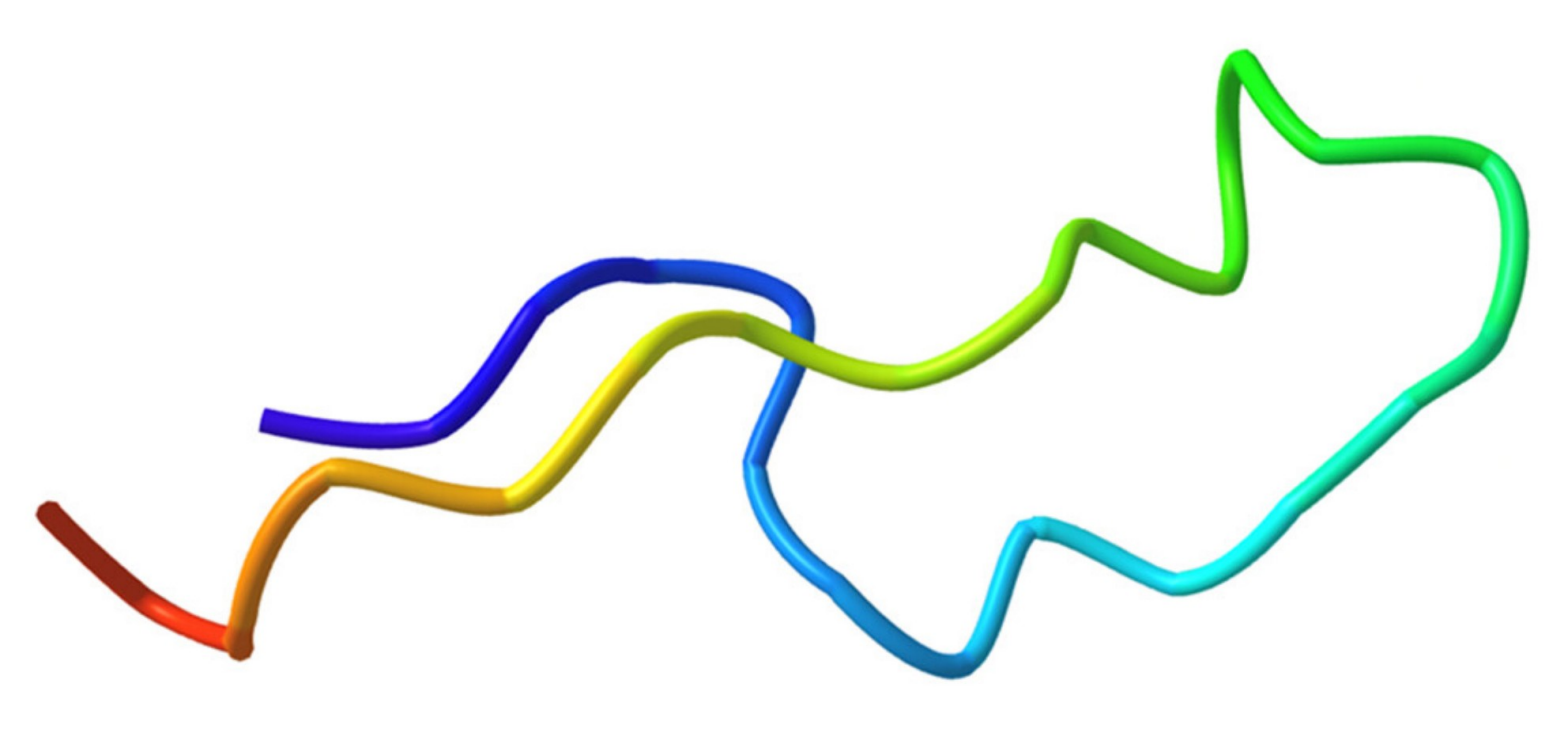
Solution‐state structure of GRASP (BMRB: 51672). Reproduced from Ref. [47].

To the same aim, Borner *et al*. used a structure‐based approach,^[47]^ starting from the model of the interactions of GDF15 with its receptor reported by Hsu *et al*.^[20]^ A first screening was performed including regions of GDF15 that are critical for forming the ternary complex and are homologous to regions within the TGFβ superfamily previously identified as potential binding sites for GDNF family receptors. This analysis redefines GDF15 as a GDNF ligand, taking into account the structure of its receptor, GFRAL, and the required recruitment of RET, a typical feature of GDNF ligands. Rather than blocking the interaction with GFRAL, the goal of the authors was the prevention of recruitment of the co‐receptor RET, and this was addressed replacing critical residues of GDF15 with bulky hydrophilic residues and incorporating sequences able to interact with metal ions in order to inhibit the interaction. Studying GFRAL binding through surface plasmon resonance, only the GRASP peptide showed binding to the target, despite having less potency compared to GDF15. GRASP is a 29‐residues peptide (TKEELIHAHADPMVLIQKTDTGVSLQTYD) in which the sequence derived from GDF15(281–298) has been combined with a zinc binding sequence (HAHA) and with two bulky hydrophilic residues (EE). The binding of the peptide to GFRAL was confirmed also *in vivo*, thus the subsequent step was the evaluation of the interaction with the ternary complex through *in silico* analysis. NMR was used to determine the solution structure of GRASP (Figure 4), in which emerged a loop region with hairpin‐like fold, and this structure was exploited to perform docking studies against the ternary complex GDF15/GFRAL/RET. GRASP showed a preferential docking site near the binding site for GDF15, as well as a further docking pocket at GDF15/RET interface.

*In silico* models suggested that GRASP interacts with multiple GFRAL sites, causing steric hindrance for RET recruitment by GDF15, thus acting as non‐competitive antagonist. Preliminary *in vivo* tests showed that GRASP colocalizes in GFRAL‐positive neurons in the AP/NTS of rats, so subsequent analysis focused on systemic and CNS administration of GRASP in rodents. As an indicator of nausea and malaise in rodents, the ingestion of non‐nutritive substances (*e. g*. kaolin) has been previously demonstrated to be a reliable translational model for evaluating the nausea‐inducing potential of pharmaceutical agents in humans.^[48]^ GRASP showed efficacy in reducing GDF15‐ and cis‐platin‐induced malaise, while minor effects were observed on food intake. This could be explained in terms of half‐life of the peptide, which is enough to guarantee effectiveness on phenomena whose manifest quickly, *i. e*., nausea, but too short to be efficacious against CINV or anorexia, which may occur at a longer time scale (days to months) thereafter.[^49^, ^50^]

These results show promising potential for a future development of a peptide, to be first tested *in vivo* and in humans, to ultimately be administrated alone or paired with other therapeutics (for instance, antiemetic agents such as Domperidone, Metoclopramide or Ondansetron), in order to mitigate the effects caused by an excessive amount of GDF15 in pathologies such as CINV or anorexia.^[47]^ However, further investigations are required to improve the pharmacological proprieties of GFRAL antagonists, and to evaluate its potential in the clinical world.

## 4 Summary and Outlook

In this review, the potential applications of derivatives of GDF15 are reported. Considering the emerging importance that this cytokine has gained since 2017 with the discovery of its receptor GFRAL, a considerable research effort has focused on the interaction between GDF15, GFRAL, and the co‐receptor RET, which all appear to be fundamental in the signalling pathway.^[20]^ Once the molecular mechanism of interaction was clarified, two potential paths opened for pharmaceutical agents, which were here summarized. The first path was the development of GFRAL agonists, species able to mimic GDF15, while the second aimed at synthesizing GFRAL antagonists, blocking the formation of the ternary complex GDF15/GFRAL/RET.

The first approach was studied to treat conditions associated to an observed deficiency of GDF15, *e. g*. obesity, or to take advantage of its activity as appetite suppressor. The design of complex proteins was required due to the poor pharmacokinetic proprieties of GDF15 itself, which hampered its therapeutic potential. In this scenario, the simplest modification was based on the use of moieties known to increase protein stability, such as Fc portions or HSA.[^26^, ^30^] Alternatively, stability could be increased *via* chemical modifications of the protein, for example exploiting N‐glycosylation to improve the solubility and/or removing or masking cleavage sites.^[34]^ Moreover, protein conjugation has been exploited to also improve pharmacodynamic parameters, coupling GDF15 with molecules able to give synergic anti‐obesity activity, *i. e*., GLP‐1.^[41]^

Two different GFRAL agonist were promoted to clinical studies. However, these fusion protein (LY3463251 and MBL949) were demonstrated not to be significantly active in humans.^[37]^ The necessity to find fusion proteins suitable for administration collides with the inherent difficulty of devising proper functionalization approach. Each part of the fusion protein seems fundamental to guarantee the maintenance of pharmacological activity for GDF15 analogues, while increasing its pharmacodynamic properties: the active moiety, which could be the entire GDF15 or, more commonly, an active portion, should be protected from proteolysis trough functionalization. The fusion moiety varies depending on the purpose of the final adduct. In any case, the fusion protein must not interfere with the active site of the GDF15 portion. To this aim, the choice of a proper linker is fundamental, since longer linkers may be more effective in reducing steric hindrance, but could cause a reduction of the production yield of the protein.^[41]^ This is also an important concern, since the expression of the designed protein is an important step and the expression yield is a parameter that may drive the design of the final product. In fact, a given fusion protein may be expressed with low yields or not be expressed at all, depending on the selected fused moiety or engineering approach.[^26^, ^34^]

Although a recent study has shown that the administration of native GDF15 is associated with nausea, vomiting and visceral malaise,^[49]^ and although studies have also shown that neutralizing GDF15 can ameliorate cancer cachexia,^[51]^ most evidence has been gathered from animal studies,[^49^, ^50^] and evidence in humans ‐ either under physiological or pathological conditions ‐ seems promising, yet limited.^[52]^

The discrepancy in effectiveness between animal models and humans may be attributed to differences in the structural properties of GDF15 analogues used in studies, for which it is often unfeasible to predict pharmacokinetic and pharmacodynamic properties either computationally or by translational perspectives alone. Indeed, long‐acting GDF15 analogues have been developed to extend the hormone's half‐life and enhance its therapeutic potential, showing robust reductions in food intake and body weight in overweight monkeys, and suggesting that structural modifications can significantly impact efficacy. In fact, biological response to GDF15 may follow a hormetic pattern, being associated with different outcomes following either short‐term pulsatile secretion or sustained and prolonged exposure.[^30^, ^53^, ^54^] Future studies could shed light on this limitation described for GFRAL agonists, potentially extending reviews to the patent literature.

On the other hand, the development of GFRAL antagonists requires a different approach. The first main difference lies in the fact that the antagonist has different and somehow simpler function: while the agonist molecule should be able to bind to GFRAL and also recruit the co‐receptor RET, in the case of antagonists, binding to GFRAL is sufficient, providing that the agent has sufficient affinity to block the interaction with native GDF15. Indeed, as described above, for the design of agonists the research focused on the whole GDF15, or on active portions of it, to guarantee the interaction with both GFRAL and RET in a ternary complex.^[21]^ Instead, in order to obtain an effective GFRAL antagonist, a short peptide sequence derived from GDF15 may be sufficient to bind to the receptor with an affinity high enough to inhibit the ternary complex formation. There are advantages in the use of synthetic peptides instead of whole proteins, *e. g*., manufacturing (not requiring *in vivo* expression), greater design flexibility (residues substitutions or functionalization are easily obtainable), and reduced risk to induce immunoresponses.^[55]^ The development of active peptide sequences, however, requires a more systematic approach, as compared to the investigation of proteins. It is mandatory to know the exact sequence of the original protein to identify binding epitope(s) and to recognize the most important residues involved in the interaction with the receptor.^[46]^ Once that one or more sequences have been found to be endowed with sufficient affinity, the interaction with the receptor may be studied through *in silico* approaches, to evaluate possible modifications to enhance the interaction, for example substituting residues or inserting amino acids, which could improve the binding with the target. Indeed, computational tools are nowadays potent enough to enable a structure‐based design starting directly from the structure of the agonist/receptor(s) complex.^[47]^ However, independently of the specific approach, the current literature clearly indicates that the use of peptides derived from GDF15 as GFRAL antagonists is still in an early phase, similarly than the overall research on GRFAL antagonists, despite recent interest in totally different molecular approaches, *e. g*., the use of antibodies.^[45]^

To be noted, however, novel molecular perspectives by peptide synthesis have emerged not only for GFRAL antagonists but also GFRAL agonists. In fact, an endogenous peptide derived from secreted GDF15 was found in mice, featuring the same functions as native GDF15: reducing food intake and body weight. This particular peptide, corresponding to region 174–185 of mouse GDF15 (pGlu‐LELRLRVAAGR‐NH_2_), presents two post‐translational modifications, N‐terminal pyroglutamylation and C‐terminal amidation (from which the name “capped peptide”) and its anorexigenic properties were demonstrated through its administration in mice.^[56]^ This interesting study, which identified an active peptide derived from mouse GDF15, could be a starting point for further investigations, looking for peptide acting as GFRAL agonist, thus a completely opposite picture, as compared to peptide antagonists discussed above.

As previously described, GFRAL belongs to the GDNF receptor family. As the literature on GFRAL ligands is, overall, still in an early phase, future studies might explore how GDF15 analogues acting as GFRAL agonists may show diverging interaction profiles with other GDNF receptors – potentially explaining their different rate of success in inducing desired effects. Future research might also investigate additional fusion molecules for GDF15 moieties, each aimed for a specific biological effect, beyond GLP‐1. For instance, fusion molecules with other neurotrophic (*e. g*. GDNF, neural cell adhesion molecule ‐ NCAM) or metabolic factors (*e. g*. ghrelin‐blockers, leptin‐inducers) could elicit better response *in vivo* to induce weight loss or appetite aversion.

In conclusion, although a substantial interest from both pharmacological companies and the scientific community, the research in the field of GDF15 and its receptor GFRAL seems to still be an almost unpaved ground ‐ full of potentials. This subject shall be further investigated from different points of view, consistently with the many different biological functions of GDF15, which suggest the possible development of different drug candidates for the treatment of diverse GDF15‐related pathologies.

## Conflict of Interests

The authors declare no conflict of interest.

## Biographical Information

*Andrea Di Santo graduated at the University of Florence (Italy) in 2022 in Chemical Sciences. His thesis, supervised by Prof. Anna Maria Papini, focused on the synthesis and characterization of antiviral peptides active against SARS‐CoV‐2. After one year of fellowship with the University of Florence, in which he studied biomolecules sustainable production methods, he is currently a PhD student in Pharmaceutical Sciences under the supervision of Prof. Paolo Rovero. His current PhD project concerns the characterization of anti‐drug antibodies in patients treated with therapeutic proteins using surface plasmon resonance*.

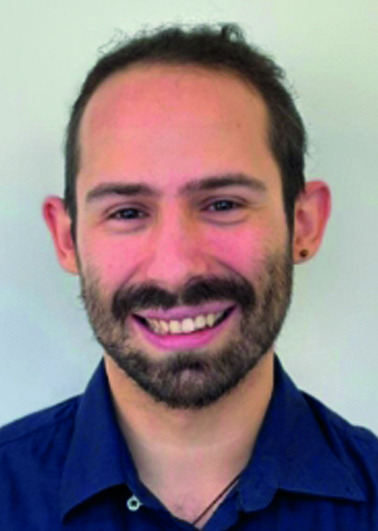

## Biographical Information

*Dr. Livio Tarchi is a dedicated medical resident currently specializing in psychiatry at the University of Florence. With a clinical focus on child and adolescent psychiatry, his professional interests center on understanding and addressing developmental and behavioral challenges, particularly those associated with eating behaviors. Dr. Tarchi is committed to providing comprehensive, evidence‐based care that supports the mental health and well‐being of young individuals during critical stages of their life*.

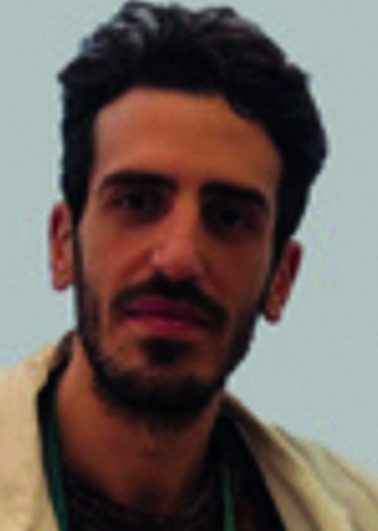

## Biographical Information

*Professor Villa is an associate professor of anaesthesiology, intensive care, and pain medicine at the University of Florence and an anaesthesiologist at Azienda Ospedaliero‐Universitaria Careggi, Florence, Italy. His expertise spans perioperative medicine, anaesthesia, intensive care, and pain management, with a particular focus on oncological care. He also serves as the medical director and safety coordinator for the Clinical Trial Unit, overseeing pharmacological phase 1 clinical studies. Additionally, he holds roles as the hospital representative for the local palliative care coordinating centre, and as the Director of the Ethics Committee at his institution*.

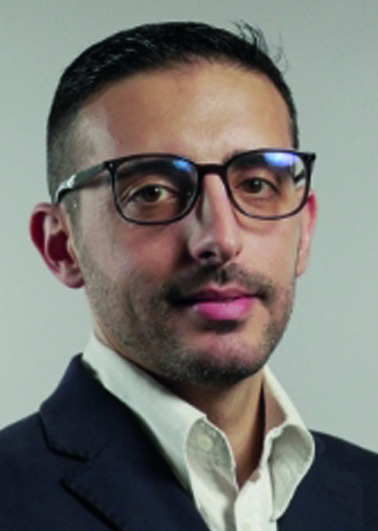

## Biographical Information

*Prof. Giovanni Castellini serves as an Associate Professor of Psychiatry at the University of Florence, Italy. A recognized authority in the field of mental health, his clinical expertise and research are devoted to the diagnosis, treatment, and prevention of eating disorders. With years of experience, Prof. Castellini has contributed significantly to the advancement of therapeutic approaches and is deeply engaged in both academic and clinical efforts to improve patient outcomes in this challenging area of psychiatry*.

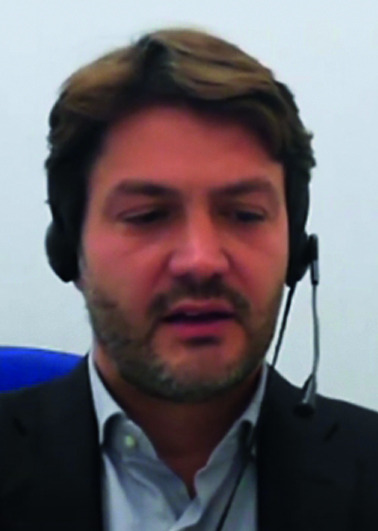

## Biographical Information

*Prof. Valdo Ricca is Full Professor of Psychiatry at the University of Florence, Italy. With an impressive academic and clinical track record, he is internationally acknowledged for his expertise in the diagnosis and treatment of eating disorders. Prof. Ricca's research interests extend to the field of psychiatric genetics, where he has made substantial contributions to understanding the genetic underpinnings of mental health conditions. As a leading figure in psychiatry, he continues to influence the discipline through his innovative research, teaching, and clinical practice*.

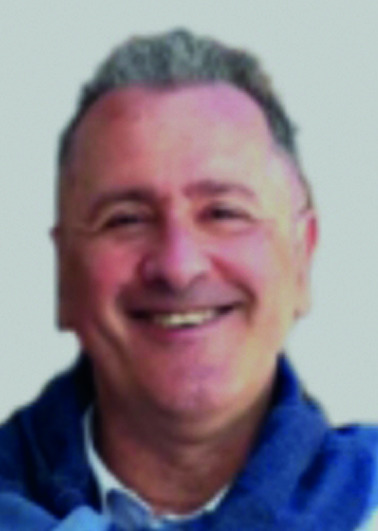

## Biographical Information

*Roberta Squecco is associate professor of Physiology at the Medical School (University of Florence). She graduated in Biological Sciences, obtained the PhD in Physiology and the Specialization in Clinical Biochemistry. In her academic career she covered various lines of research working on skeletal muscle (excitation‐contraction coupling, damage, atrophy and regeneration); on stem cell bioelectrical properties; on the role of peptide hormones on the circuits controlling the hunger‐satiety cycle, focusing on the modulation of gastrointestinal tract motility. She published 77 papers in international journals. She is member of the Italian Physiological Society, Interuniversity Institute of Myology and Stem Cell Research Italy*.

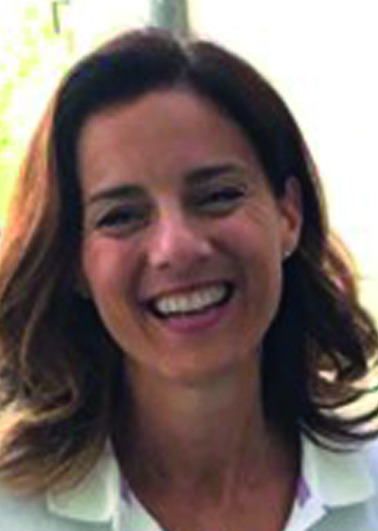

## Biographical Information

*Anna Maria Papini is Full Professor of Bioorganic Chemistry at the Department of Chemistry, University of Florence, where she coordinates the Interdepartmental Research Unit of Peptide and Protein Chemistry and Biology. She completed her PhD in 1990 under the supervision of L. Moroder (Department of Peptide Chemistry, MPI for Biochemistry). As Laureate of the “Chaire d'Excellence” (French ANR 2009–2020), she founded PeptLab@UCP at CY Cergy Paris University, producing active peptide ingredients at a large scale. She founded and was CSO of Toscana Biomarkers (2007–2014). She co‐chaired 14th IPS/37th EPS (Florence, 25–31 August 2024)*.

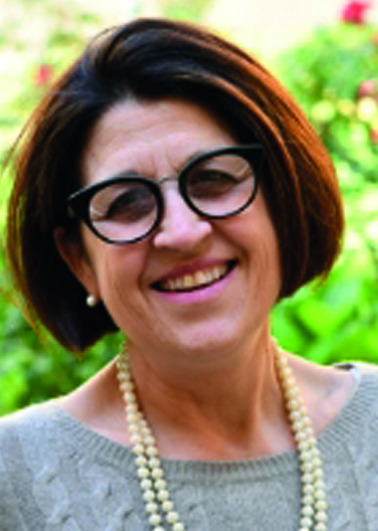

## Biographical Information

*Feliciana Real Fernandez is a researcher at the Institute of Chemistry of Organometallic Compounds ‐ National Research Council of Italy (ICCOM‐CNR). Her research interests concern the design, synthesis and biological characterization of peptides and their use as antigenic probes in immunoenzymatic assays to study peptide/protein‐antibodies interaction. She has developed a surface plasmon resonance‐based methodology to identify and quantify anti‐adalimumab antibodies directly in treated patients’ sera. Other activities include the quantification of endocrine disrupting chemicals in biological fluids. She is coauthor of over 45 scientific publications in international journals and 1 patent*.

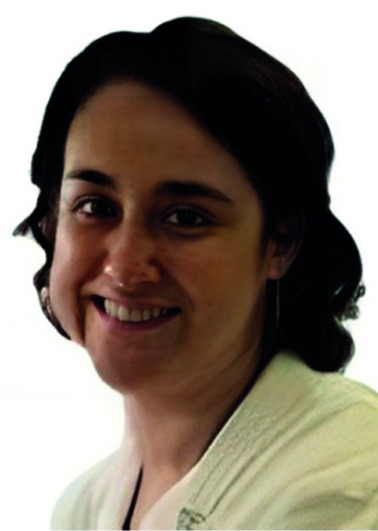

## Biographical Information

*Paolo Rovero is full professor of pharmaceutical chemistry at the Medical School of the University of Florence. Since 1985, his research interests concern the design, synthesis and biological characterization of peptides of pharmaceutical, cosmeceutical and biotechnological interest. After specializing in peptide science in Sherbrooke, Canada (1985–86), his career started in a leading Italian pharmaceutical company (Menarini, Florence, Italy 1986–1991) and continued in various academic contexts. He is co‐author of over 270 scientific publications in international journals and 25 patents of pharmaceutical relevance. He is Editor‐in‐Chief of the Journal of Peptide Science, published by the European Peptide Society*.